# TOGETHER IN CARE: ELEVATING THE IMPORTANCE OF FAMILY AND PAID CAREGIVERS

**DOI:** 10.1093/geroni/igae098.1693

**Published:** 2024-12-31

**Authors:** Jessica King, Kezia Scales, Amy Robins, Toni Gingerelli

**Affiliations:** PHI, New York, New York, United States; PHI, New York, New York, United States; PHI, New York, New York, United States; National Alliance for Caregiving, Washington, District of Columbia, United States

## Abstract

In 2023, PHI and the National Alliance for Caregiving (NAC) launched “Together In Care: The Direct Care Worker and Family Caregiver Initiative” with funding from the John A. Hartford Foundation. The goal of the initiative is to promote policy changes that address the interrelated needs of family caregivers and paid caregivers in the context of long-term care for older adults and people with disabilities, and particularly in home and community-based services (HCBS). Priority topics for the initiative include improving care team integration for family members and paid caregivers; advancing self-direction options and supports; and securing funding for new research on the underexplored nexus of paid and unpaid care. This presentation will begin by describing the conceptual rationale for focusing on the interconnection and overlap between paid and family caregivers, referencing the limited extant literature on this topic. We will then discuss the methodology and evaluation of our efforts to develop a shared caregiving policy agenda across diverse partners — who include federal and state officials, national experts and movement leaders, and long-term care providers — highlighting successes and lessons learned. The presentation will underscore the importance of using evaluation research methods to inform and assess the implementation and impact of complex policy advocacy interventions; share practical approaches and methods; and end with a discussion of the extensive research gaps and opportunities that have been identified through this innovative initiative.

